# Dataset for multidimensional assessment to incentivise decentralised energy investments in Sub-Saharan Africa

**DOI:** 10.1016/j.dib.2021.107265

**Published:** 2021-07-14

**Authors:** A. Bender, M. Moner-Girona, W. Becker, K. Bódis, S. Szabó, A.G. Kararach, L.D. Anadon

**Affiliations:** a University of Cambridge, Centre for Environment, Energy and Natural Resource Governance, Cambridge, United Kingdom; b Victoria University of Wellington, Geography, Environment and Earth Sciences, New Zealand; c European Commission, Joint Research Centre (JRC), Ispra, Italy; d Institute of Advanced Studies (iASK), Kőszeg, Hungary; e European Institute of Innovation and Technology, Budapest, Hungary; f African Development Bank Group, South Africa

**Keywords:** Africa, Photovoltaic, Energy access, Mini-grid, Multidimensional approach, Energy development policy, Energy investments, Spatial analysis, Composite indicator

## Abstract

In this data article, we present datasets from the construction of a composite indicator, the Photovoltaic Decentralised Energy Investment (PV-DEI) index, presented in detail in [1]. This article consists of a comprehensive energy-related data collected in practice from several sources, and from the outputs of the methodology described in [1]. The PV-DEI was designed and developed to measure the multidimensional factors that currently direct decentralised renewable energy investments. The PV-DEI index includes 52 indicators and was constructed because factors stimulating investment cannot be captured by a single indicator, e.g. competitiveness, affordability, or governance [1]. The PV-DEI index was built in alignment with a theoretical framework guided by an extensive review of the literature surrounding investment in decentralised Photovoltaic (PV), which led to the selection of its indicators. The structure of the PV-DEI was evaluated for its soundness using correlational assessments and principal component analyses (PCA). The raw data provided in this article can enable stakeholders to focus on specific country indicators, and how scores on these indicators contributed to a countries overall rank within the PV-DEI index. The data can be used to weight indicators depending on the specifications of several different stakeholders (such as NGOs, private sector or international institutions).

## Specifications Table

**Table:** 

Subject	Energy:
Specific subject area	Renewable Energy, Sustainability and the Environment
Type of data	Table Figure Spreadsheet
How data were acquired	Queried from Open Data portals, systematically joined and cleaned. Compiled based on a comprehensive horizon-scanning of data sources that are processed for a composite indicator
Data format	Formatted data (Table 1–10 and Tables A1-A10 in Appendix A); Processed and analysed data (Fig. 1, Appendix B Tables B1-B4).
Parameters for data collection	The rationale for collecting the variables was to select indicators related to the economic, energy, environmental, financial and institutional frameworks of Sub-Sahara African countries.
Description of data collection	Data is collected by systematic queries. Compiled and formatted data compilations are utilized for data processing and analyses in the context of the research work.
Data source location	Secondary data in supplementary material Primary data sources: Joint Resarch Centre (European Commission), PVGIS JRC-European Commission. PVGIS 2020. http://re.jrc.ec.europa.eu/pvgis (accessed February 2, 2020) [2] Huld T, Moner-Girona M, Kriston A. Geospatial Analysis of Photovoltaic Mini-Grid System Performance. Energies 2017;10:218. https://doi.org/10.3390/en10020218. University of Denmark (DTU), The World Bank Group. Global Wind Atlas 2017. https://globalwindatlas.info/(accessed July 20, 2019) [3] Renewable Fuel Association. Fuel Ethanol Trade Measurements and Conversions 2015 [4] Szabo et al. https://doi.org/10.1088/1748-9326/6/3/034002[5], NASA Earth Observatory. Earth at Night 2019. https://earthobservatory.nasa.gov/ (accessed May 7, 2019) [6], European Commission Joint Research Centre (JRC). Global Human Settlement Layer (2019). Ghs_pop2019 @ghslJrcEcEuropaEu n.d. https://ghsl.jrc.ec.europa.eu/ghs_pop2019.php (accessed July 7, 2019) [7] The World Bank Group. World Bank Indicators 2019. https://data.worldbank.org/ (accessed June 6, 2019) [8] World Economic Forum. The Global Human Capital Report 2017. Geneva, Switzerland: 2017 [9] The World Bank Group, The International Finance Coorporation (IFC). Lighting Africa 2019. https://www.lightingafrica.org/ (accessed June 6, 2019) [10] The World Health Organization (WHO). Global Ambient Air Quality Database 2018. https://www.who.int/airpollution/data/en / (accessed June 6, 2019 [11]
	International Monetary Fund. International Financial Statistics and data 2019. https://www.imf.org/en/Data[12] The Shift Project. The Shift Project 2019. https://theshiftdataportal.org/ (accessed June 6, 2019) [13] United Nations Development Programme. Human Development Report 2016: Human Development for Everyone. 2016. https://doi.org/eISBN:978-92-1-060036- [14] International Energy Agency. World Energy Outlook 2017. Paris, France: OECD/IEA; 2017. http://www.iea.org/publications/freepublications/publication/WEB_WorldEnergyOutlook2015ExecutiveSummaryEnglishFinal.pdf[15] IFPRI, (WHH) W, Worldwide C. 2016 Global Hunger Index Data 2016. https://doi.org/10.7910/DVN/LU8KRU[16] World Bank Development Research Group, Institution NRGI (NRGI) and B. Worldwide Governance Indicators 2018. https://info.worldbank.org/governance/wgi/ (accessed June 6, 2019)[17]. World Justice Project. Rule of Law Index. Washington, US: 2018. https://doi.org/10.4135/9781483381503.n1030[18] Ibrahim Index of African Governance. Mo Ibrahim Foundation. Ibrahim Index of African Governance. London, UK: Mo Ibrahim Foundation; 2015. [19] Bloomberg New Energy Finance. Climatescope 2018. Clim 2018 2019. http://global-climatescope.org/ (accessed June 6, 2019) [20] Global Competitiveness Report The Global Competitiveness Report 2017–2018. World Economic Forum [21] IRENA. Global atlas for renewable energy n.d. https://irena.masdar.ac.ae/gallery/#gallery (accessed January 1, 2017). [22] Szabó S, Pinedo Pascua I, Puig D, Moner-Girona M, Negre M, Huld T, et al. Mapping of affordability levels for photovoltaic-based electricity generation in the solar belt of sub-Saharan Africa, East Asia and South Asia. Nat Sci Reports 2021;11. https://doi.org/10.1038/s41598-021-82638-x. [23]
Data accessibility	https://data.jrc.ec.europa.eu/collection/id-0076
Related research article	Moner-Girona, M., Bender, A., Becker, W., Bódis, K., Szabó, S., Kararach, A.G., and Anadon, L.D, A multidimensional high-resolution assessment approach to boost decentralised energy investments in Sub-Saharan Africa, Renew Sustain Energy Rev, https://doi.org/10.1016/j.rser.2021.111282

## Value of the Data

• The data is suitable for constructing a composite indicator for directing/informing decentralised renewable energy investments in Sub-Saharan Africa
• The datasets integrate technological, environmental, social, political and financial indicators for decision support
• The raw data is made publicly available, and is a unique resource which allows stakeholders to examine the specific situations of countries, and make comparisons in detail
• Different weights can be applied to the raw data to enable stakeholders to change the importance they place on certain indicators depending on their own specifications (such as from a NGO, private sector, international institutions or other perspective)

## 1 Data Description

**Fig. 1:**
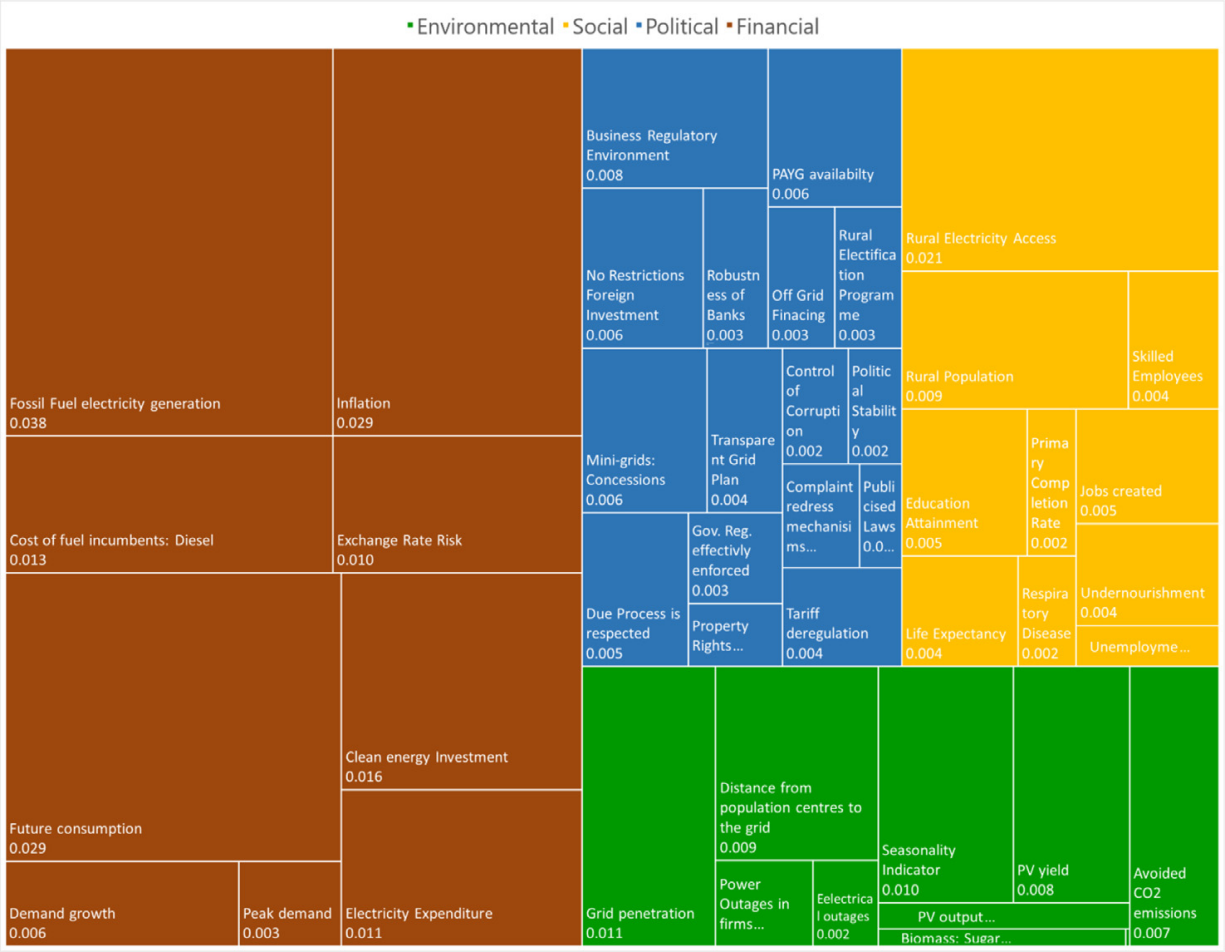
**Break down of the PV-DEI index for Congo (last ranking) under a private sector approach**: 4 main dimensions (Environmental, Social, Political and Financial), 18 pillars, 43 sub-pillars and 52 indicators. The size of the coloured squares represents the overall weights of the dimension and the size of each square the weights of the individual indicator.

This article contains the data compilations for design and development of the PV-Decentralised Energy Investment (PV-DEI) Index for Sub-Sahara African countries. The PV-DEI Index is built in 4 main dimensions (Environmental, Social, Political and Financial), 18 pillars, 43 sub-pillars and 52 indicators. In Fig. 1 the size of the coloured square represents the overall weight of a dimension, and the size of each square represents the weight of an individual indicator. The description of the data sets are provided in the data tables for the main indicators of each dimension in this article, while raw data are provided in table in the Supplementary Information. The original research article [1] describes the analysis and methodology used to create the PV-DEI Index.

Tables SI.1-SI.4 Show the methodology employed to gather the raw data used to compose the PV-DEI Index for the four dimensions: Environmental (Tables SI.1), Social (Tables SI.2), Political (Tables SI.3), and Financial (Tables SI.4).

Table SI.5 shows the weights used for the private sector approach.

Table SI.6 contains the original data used as inputs in the COIN tool [24] without data treatment.

Table SI.7 contains the data after winzorization.

Table SI.8 Contains the results of COIN tool after calculating the correlations between indicators (Pearson coefficients r) taking into account the direction of effects.

Figure SI.9 and SI.10 contains the datasets after MICE and FOREST imputation respectively.

Table 1 summarises the classification, source, year and description of the 52 indicators that build the PV-DEI index.

**Table 1:** Composite Indicators for the PV-DEI Index.

Dimension		Pillar	Indicator Name		Data Source	Year	Description:	Indicator direction
Environmental si.1	p.01	Resources	Ind.01	PV output- Country Average	PVGIS [2], Huld et al. [25]	2019	The PV output (kWh/kW_p_) represents the theoretical average electricity production per year per kW_p_ installed. Its importance is evidenced by its universal inclusion in modelling papers. PV output directly impacts the amount of energy that can be produced and the levelised cost of electrcity (LCOE). Thus, like proximity to the current-grid it represents a hard limit to the economic competitiveness of decentralised solar-PV.	Positive
			Ind.02	PV output-Spatial variability	PVGIS [2], Huld et al. [25]	2019	Spatial Standard Deviation in PV output (kWh/kW_p_) The greater the deviation in solar potential throughout the country territory, once the system is optimised by the best location in terms of PV output the more intermittent the reliable power supply becomes along the territory.	Negative
			Ind.03	Seasonality Indicator	PVGIS [2], Huld et al. [25]	2019	Standard deviation in PV output across months of the year In the evaluation by Huld et al. [2,25], seasonality was the main determinant of the battery size required for decentralised solar-PV systems. The greater the deviation in solar potential throughout the year the less reliable the power supply becomes year round, this leads to greater reliance on expensive battery storage and/or a larger PV system to deliver the same amount of electricity	Negative
			in.04	Wind resource Endowment	DTU [3], IRENA [22]	2014	Wind resource Endowment (TWh per year) for each country, [3,22]. Rather than competing with solar, wind resources can be used in conjunction with solar PV to increase the reliability of power supply by utilising two rather than one intermittent supplies	Positive
			in.05	Biomass resource potential	IRENA [22], RFA [4]	2014	The biomass resource potential IRENA [22] was calculated by converting data on the Million litres of ethanol from sugar cane (Total land area, no restrictions, rain fed, > 2 tonnes per hectare) present in a country into GWh per year using the following steps: 1. Converts ethanol energy into equivalent oil barrels [4]. 2. Converts oil barrels into GWh. It can be used to augment solar power supply by providing power to back up generators etc.… that can be used seasonably when solar is not available	Positive
	p.02	Existing Infrastructure	ind.06	Grid Penetration	Szabo et al. [5], NASA [6], JRC-GHSL [26]	2019	In the model used to calculate grid penetration [5] decentralised Solar-PV was unable to compete with grid-connected incumbents in areas proximate to the current grid due to infilling and cost-competitive extension of the existing infrastructure. Thus, this exclusion zone represented a hard limit where renewable technologies were unlikely to be economically competitive. The extent of the current grid also indicates an established reliance on incumbent technologies, which may be challenging to displace for sociocultural reasons. Grid penetration is aggregated at the country level and is the percentage of a country population living close to the existing electricity grid (inside 5 km inclusion zone) or/and zones where there is already light. Calculated using the GIS model of the electricity grid within SSA countries and establishing 5Km buffer zones around where the grid exists and/or where nightlight data indicates that the grid exists (methods section). The calculating the number of people residing inside this exclusion zone and dividing by the total population in the country. This is negatively weighted as the more people inside the existing grid zone the less relevant decentralised technologies are compared to expanding last mile grid coverage, and fewer rural populations need decentralised technologies	Negative
			ind.07	Distance from population settlements to the grid	Szabo et al. [5], NASA [6], JRC-GHSL [26]	2019	Distance from population centres to the grid. The locations of population centres JRC-GHSL [26] without access to electricity were established using the population out of the grid buffer and without nightlight. Weighted positively: The further population centres are from the grid the more expensive it will be for grid electrification to reach them and the more important decentralised solar-PV options will be.	Positive
			ind.08	Power Outages in firms in a Typical Month	World Bank [8]	(⁎⁎)	The less reliable the incumbent energy solution is the more important decentralised solutions that can provide reliable power will be (e.g. solar- PV with battery storage)	Positive
			ind.09	Value lost due to electrical outages	World Bank [8]	(⁎⁎)	The less reliable the incumbent energy solution is the more important decentralised solutions that can provide reliable power will be (e.g. solar- PV with battery storage)	Positive
	p.03	Avoided Emissions	ind.10	CO_2_ emissions avoided by the PV mini-grid directly related to kwh produced	JRC model	2019	CO_2_ emissions avoided by the PV mini-grid directly related to kwh produced C0_2_ emissions avoided per year by the PV-mini grid instead of diesel mini-grid; data taken from model indicator per pixel (tCO_2_)	Positive
Social si.2	p.04	Accessibility	ind.11	Rural Population	World Bank [8]	2017	Rural population as a percentage of the total population. The larger the rural population within a country the greater the potential for decentralised solar PV solution to help a significant number of people (even if electrified current rural electricity solutions tend to be expensive, sometimes dangerous and often unreliable)	Positive
			ind.12	Rural Access to Electricity	World Bank [8]	2017	Rural Access to electricity as a percentage of the rural population. This indicator captures the sub-section of the rural population who already have access to some electricity and are therefore there is less market potential for decentralised energy solutions	Negative
			ind.13	Perceived availability of Skilled Employees	Human Capital Index World Economic Forum (WEF) [9]	2017	Reflects the human capital endemic in a country, which is beneficial both for establishing a business and enabling novel technologies to diffuse deeper and winder within communities The indicator was calculated by the WEF using an executive opinion survey [9].	Positive
			ind.14	Consumer Knowledge - Lighting Africa Consumer Awareness Campaign	Lighting Africa [10]	2019	Calculated using categorical data. Data available on the ‘Lighting Africa Consumer Awareness Campaign’. If there was an ‘Established Consumer Awareness Campaign by Lighting Africa’ the country scored 3 points, if there was a ‘Recently Initiated/Imminent Consumer Awareness Campaign by Lighting Africa’ the country scored 2 points and if there had been ‘Lighting Africa Involvement in ECOWAS Regional off-grid electrification project’ the country scored 1 point. The aim of this indicator was to capture existing knowledge about decentralised solar PV energy provision, as this reflect the likelihood of successful integration, utilisation and diffusion of the technology into communities.	Positive
	p.05	Impacts	ind.15	Life Expectancy at Birth	World Bank [8]	2017	Life expectancy at Birth, country average data. This indicator was measuring the anticipated health impact of bringing power through decentralised solar-PV, therefore the worse the life expectancy the higher is the impact of bringing electricity.	Negative
			ind.16	Respiratory Disease Incidence	WHO [11]	2018	Respiratory disease incidence per 100,000 population. The higher the incidence of respiratory disease the more beneficial decentralised renewable energy solutions may be, both in terms of electrifying health centres that target respiratory disease and in terms of replacing dirty fuels known to cause respiratory disease	Positive
			ind.17	Education Attainment - Harmonized Test scores	World Bank [8]	2017	Education Attainment measured using average harmonized test scores within countries. This indicator was measuring the anticipated educational impact of bringing electricity to communities; therefore, the impact of binging electricity will be higher in communities where the educational is low.	Negative
			ind.18	Primary Completion Rate	World Bank [8]	(^⁎⁎⁎^)	Primary Completion rate weighted negatively for the same reasons as above. Used in conjunction with the indicator above - in case high test scores were mediated by a decline in participation of less able students due to lower completion.	Negative
			ind.19	Gender. Unemployment Rate - Female to Male Ratio	UNDP Human development Indicators [14]	2017	Ratio of the percentage of the female labour force population ages 15 and older, which is not in paid employment or self-employed but, is available for work and is actively seeking paid employment or self-employment to the percentage of the male labour force population ages 15 and older in the same status. A higher score reflects greater female emancipation within the labour market, and thus a lower potential impact of electricity provision for improving female liberty.	Negative
			ind.20	Estimated number of jobs created	JRC-GHSL [26], OECD [ref].	2019	Estimated potential number of jobs created directly related to the deployment of PV mini-grids: The greater the percentage of people within a country who can gain employment from solar-PV establishment the better for the local economy (growth hypothesis). This was calculated using data on the total MWh of electricity output anticipated if the total number of potential mini-grids were established within each country and the employment factors come from OECD [ref].	Positive
			ind.21	Prevalence of Undernourishment	International Food Policy Research Institute [16]	2014–2016	Prevalence of undernourishment as a percentage of the population. The greater the prevalence of undernourishment the greater the potential for nutritional improvements from solar-PV deployment, (which can be used to make agricultural practices more efficient and refrigerate food produce to store for longer).	Positive
Political si.3	p.06	Political Environment	Ind.22	Political Stability and Absence of Violence	World Bank Worldwide Governance Indicators [17]	2018	Stability and Absence of Violence/Terrorism measures perceptions of the likelihood of political instability and/or politically motivated violence, including terrorism.	Positive
			Ind.23	Control of Corruption	World Bank Worldwide Governance Indicators [17]	2018	Corruption defined as the risk that individuals/companies will face bribery or other corrupt practices to carry out business, from securing major contracts to being allowed to import/export a small product or obtain everyday paperwork. This threatens a company's ability to operate in a country, or opens it up to legal or regulatory penalties and reputational damage.	Positive
			Ind.24	Publicised Laws	World Justice Project [18]	2019	Publicised laws, data was taken directly from the World Justice Project [18]: Open Governance indicator category, from the sub-indicator titled ‘Publicized Laws and Government Data’. This measured: ‘Whether basic laws and information on legal rights are publicly available, presented in plain language, and made accessible in all languages. It also measures the quality and accessibility of information published by the government in print or online, and whether administrative regulations, drafts of legislation, and high court decisions are made accessible to the public in a timely manner’.	Positive
			Ind.25	Complaint redress mechanisms	World Justice Project [18]	2019	Complaint Redress Mechanism, data was taken directly from the World Justice Project: Open Governance indicator category, from the sub-indicator titled ‘Complaint Mechanism’. This measured ‘whether people are able to bring specific complaints to the government about the provision of public services or the performance of government officers in carrying out their legal duties in practice, and how government officials respond to such complaints’.	Positive
			Ind.26	Government regulation effectively enforced	World Justice Project [18]	2019	Government regulation effectively enforced, data was taken directly from the World Justice Project: Regulatory Enforcement indicator category, from the sub-indicator with the same title. This measured: ‘whether government regulations, such as labour, environmental, public health, commercial, and consumer protection regulations are effectively enforced’.	Positive
			Ind.27	Due Process is respected	World Justice Project [18]	2019	Due Process is respected, data was taken directly from the World Justice Project: Regulatory Enforcement indicator category, from the sub-indicator with the title ‘Due process is respected in administrative proceedings. This measured ‘whether the due process of law is respected in administrative proceedings conducted by national and local authorities in issue areas such as the environment, taxes, and labour.	Positive
			Ind.28	Property Rights: No unlawful expropriation without adequate compensation	World Justice Project [18]	2019	Property Rights: No unlawful expropriation without adequate compensation, data was taken directly from the World Justice Project: Regulatory Enforcement indicator, from the sub-indicator titled: ‘The government does not expropriate without lawful process & adequate compensation.’ This measured ‘whether the government respects the property rights of people and corporations, refrains from the illegal seizure of private property, and provides adequate compensation when property is legally expropriated.’	Positive
			ind.29	Business Regulatory Environment	Ibrahim Index of African Governance IIAG [19]	2017	Business Regulatory Environment, data was taken directly from the Ibrahim Index of African Governance [19] within the ‘Business Environment’ category. The 2018 IIAG is calculated using data from 35 independent African and global data sources.	Positive
			ind.30	Project Development Barriers	ClimateScope [20]	2018	Categorical data. Project development Barriers, this data was taken directly from ClimateScope [20] within their Fundamentals’ category. This indicator ‘rewards countries where developing renewables projects is frictionless.	Negative
			ind.31	Absence of Restrictions on Foreign Investment	Ibrahim Index of African Governance [19]	2017	Absence of Restrictions on Foreign Investment, data was taken directly from Ibrahim Index of African Governance IIAG [19]’ within the ‘Business Environment’ category.	Positive
			ind.32	Robustness of Banks	WEF Global Competitiveness Report [21]	2018	Robustness of Banks, this data was taken directly from the World Economic Forum's Global Competitiveness Report 2018 [21] from the 9th pillar ‘Financial System’ from the indicator titled: Soundness of Banks. This was calculated using ‘responses to the survey question, “In your country, how do you assess the soundness of banks?” [1 = extremely low banks may require recapitalization; 7 = extremely high banks are generally healthy with sound balance sheets]	Positive
	p.07 Decentralised Energy Market	Ind.33	Light Handed regulatory framework	ClimateScope [20]	2018		Categorical data. Light Handed regulatory framework, this data was taken directly from ClimateScope2018, from within their ‘Opportunities’ category. This indicator ‘rewards countries where the regulatory framework for developing off-grid projects has the least red tape.’	Positive
		Ind.34	Off Grid Financing Facilities	ClimateScope [20]	2018		Categorical data. Off-grid financing facilities, this data was taken directly from ClimateScope2018, from within their ‘Opportunities’ category. This indicator ‘rewards countries where public or private financing facilities for off grid renewables projects are available.’	Positive
		Ind.35	Rural Electrification Programme	ClimateScope [20]	2018		Categorical data. Rural electrification program, this data was taken directly from ClimateScope2018, from within their ‘Opportunities’ category. This indicator ‘rewards countries where a detailed rural electrification program is in place.	Positive
		Ind.36	Pay As You Go (PAYG) availability	ClimateScope [20]	2018		Categorical data. PAYG availability, this data was taken directly from ClimateScope2018, from within their ‘Fundamentals’ category. This indicator ‘rewards countries where pay-as-you go solar technology is available.’	Positive
		Ind.37	Transparent Grid Extension Plan	ClimateScope [20]	2018		Categorical data. Data from ‘Fundamentals’ in ClimateScope2018 [20]. This indicator ‘awards points to countries where electricity grid transmission plans can be accessed by energy sector stakeholders.	Positive
		Ind.38	Mini-grids concessions,	ClimateScope [20]	2018		Categorical data. Data from Fundamentals’ category in ClimateScope2018 [20].. This indicator ‘rewards countries where the regulator awards developers off-grid electrification concessions in which they can operate as a monopoly.’	Positive
		Ind.39	Off-grid energy access target	ClimateScope [20]	2018		Categorical data. Off-grid energy access target, data from ClimateScope2018 [20], ‘Fundamentals’ category. This indicator ‘rewards countries for having an energy access target that recognizes the role off-grid technologies can play in improving electrification levels.’	Positive
		Ind.40	Tariff deregulation	ClimateScope [20]	2018		Categorical data. Tariff deregulation, this data was taken directly from ClimateScope2018, from within their ‘Fundamentals’ category. This indicator ‘rewards countries where off-grid developers can structure the tariffs they charge for their electricity themselves.’	Positive
		Ind.41	Tax/ Duty Reductions	ClimateScope [20], IRENA	2018		Categorical data. Tax / duty reductions data from ClimateScope2018, from within their ‘Opportunities’ category. This indicator ‘rewards countries where renewables benefit of reductions in tax and duties.	Positive
Financial si.4	p.08	Financial Risk	Ind.42	Weighted Average Cost of Capital (WACC)	Ondraczek et al. [27]	2014	In Ondraczek et al. [27] results that in high financing costs were a critical barrier to investment in LEDC's. High financing cost were associated with lower likelihood of investment in solar-PV projects.	Negative
			Ind.43	Inflation	IMF [12]	2015–2017	Inflation, data from the Wold Bank indicator: ‘Inflation, consumer prices (annual%)’ from the ‘International Monetary Fund, International Financial Statistics and data files’.	Negative
			Ind.44	Exchange Rate Risk Standard Deviation in Exchange rate between 2013 and 2018	World Bank [8]	2013–2018	Exchange Rate Risk measured the volatility in the exchange rate within each country and therefore the risk that currency could change in value prior to conversion. This was achieved by measuring the Standard Deviation in Exchange rate between 2013 and 2018 using data from the World Bank indicator: ‘Official exchange rate (LCU per US$, period average)’..	Negative
			Ind.45	Cost of fuel incumbents: Diesel	World Bank [8]	2016	Data from the ‘Pump price for diesel fuel (US$ per litre)’ World Bank indicator. The more expensive the incumbent the more competitive solar-PV power provision would be.	Positive
			Ind.46	Current electricity generation from coal, oil and gas	The Shift Project [13]	2014	Indicator intended to measure the entrenched reliance on fossil fuels already established within a country. Data from the ‘Shift Project’ dataset: ‘Breakdown of Electricity Generation by Energy Source.’ From this, the datasets of the 3 major fossil fuel energy sources: Coal, Oil and Gas, were combined. The more entrench the fossil fuel system the more impenetrable the market for renewable energy technologies's, and the more likely grid-expansion is to be the lower cost option/ subsided option	Negative
	p.09	Market Size	Ind.47	Electricity Expenditure Per Day	World Bank [8], JRC	2019	Data from ongoing JRC research. The higher the current expenditure the more likely solar-PV is to be competitive/ provide a lower cost alternative	Positive
			Ind.48	10 year electricity demand growth projections	ClimateScope [20]	2018	Data from ‘Opportunities’ category [20]. This indicator rewards countries where Bloomberg NEF projected electricity demand growth is the highest.	Positive
			Ind.49	Growth Rate of Peak demand 5 year rolling average	ClimateScope [20]	2018	Data from ‘Opportunities’ category [20]. This indicator ‘rewards countries where historic peak electricity demand growth (5 year rolling average) is the highest.’	Positive
			Ind.50	Future consumption	Moner-Girona et al. [1]	2019	Future consumption based on model estimated potential (see section methods [1])	Positive
	p.10	Experience in the sector	Ind.51	Clean energy Investment $	ClimateScope [20]	2018	Data from ‘Experience’ category [20]. This indicator rewards countries where historic clean energy investment is the highest (levelized against GDP).’	Positive
			Ind.52	Foreign investment in Clean energy	ClimateScope [20]	2018	Data from ‘Experience’ category [20]. This indicator rewards countries where the share of foreign investment in renewables asset finance is the highest.	Positive

* i.e. total number at country level - Country Score - Country Average Value -% of land/population - per capita.

⁎⁎ Most recent reported figure since 2010.

Table 2 gathers for each country: the market size for decentralised energy options (potential new costumers, total investment costs needs, average levelised cost of electricity and total avoided CO2 emissions.

**Table 2:** **Market size for decentralised options, total investment costs needs, average levelised cost of electricity (LCOE) and total avoided CO_2_ emissions.** The market size represents the amount of population living in areas favourable to decentralised energy options (more than 5 km distant of the existing grid and no lighting). The market size is split for the two main options: PV mini-grids (higher density of population) and stand-alone systems (more dispersed population). The total investment costs (NPV) are calculated aggregating the total cost of decentralised energy options taking into account the optimised size of the system for each location and specific load consumption per decentralised system zone (aggregation of cells), the density of population and the economy of scales (lower upfront cost for larger systems). The LCOE is calculated as an average of the LCOE values per country taking only the areas covered by decentralised options. The avoided CO2 emissions are calculated comparing with emissions of diesel generators. The table is sorted by mini-grid market size, with the colours in the left column indicating the overall ranking group in the PV-DEI index (from green most favourable to red least favourable).

Fig. 1 displays the breakdown of the PV-DEI index for Congo as an example of the weight of each dimension and indicators.

**Fig. 2:**
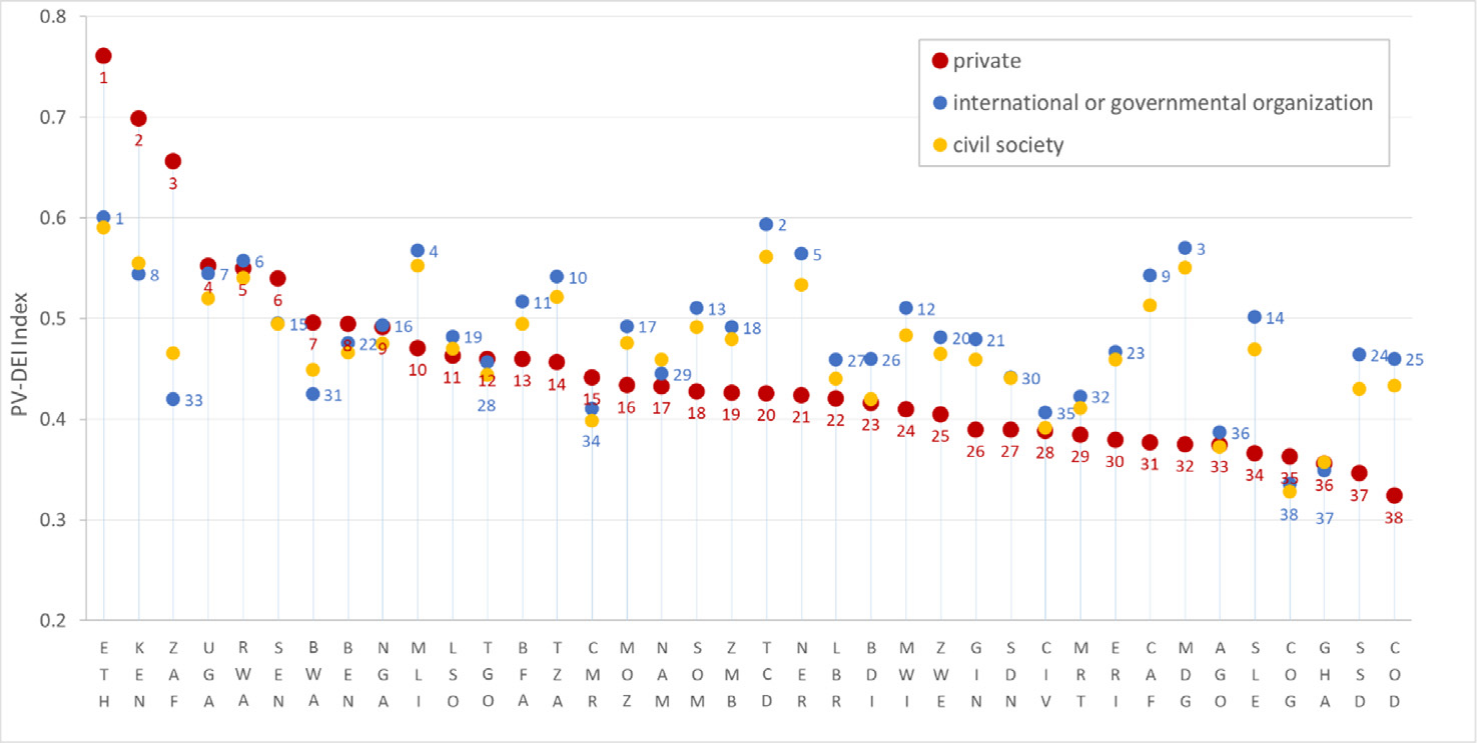
**PV-DEI index variability under three different perspectives private sector, civil society, and international donors:** The baseline scenario is determined by the PCA analysis.

Fig. 2 shows the PV-DEI index variability under three different perspectives private sector, civil society, and international donors: The baseline scenario is determined by the Principle Component Analysis.

**Fig. 3:**
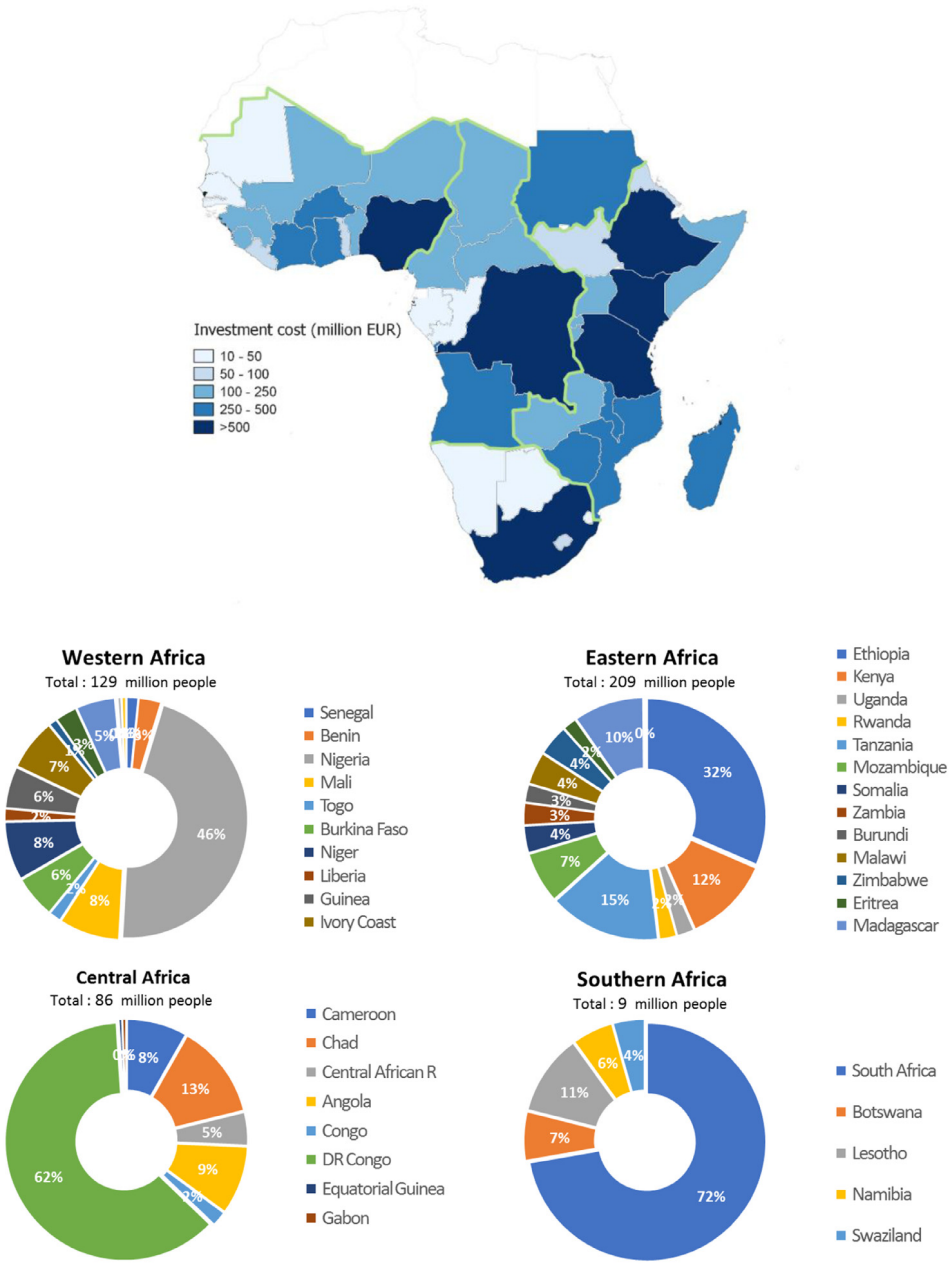
Cumulative investments (EUR million) per country for 20 years on PV decentralised options (dark blue for higher total amount of investments). B¬The pie charts indicate the cumulative share of market size (new potential costumers) for each African region.

Fig. 3 depicts the overall investment costs (NPV), are the total amount of investment in PV decentralised option per country.

Fig. 5. A displays Correlational assessments carried out in the COIN tool on the non-imputed data sets B ¬ displays Correlational assessments carried out in the COIN tool showing results from one of the 5 MICE imputed data sets C¬ displays Correlational assessments carried out in the COIN tool on the MissForest imputed data sets

Fig. 6. A ¬ shows PV-DEI scores calculated using the pooled results of the 5 MICE() imputed datasets .B ¬ shows PV-DEI Scores calculated using the MissForest() imputed data

Fig. 1 shows the breakdown of the PV-DEI index for Congo as an example of the weight of each dimension and sub-indicators.

Fig. 2 illustrates the sensitivity analysis investigating whether the scores and/or their associated inferences are robust with respect to changes in the weighting systems indicative of different stakeholder perspectives [28,29].

Fig. 3 depicts the estimated required investment needs for decentralised solar-PV in a country. These represent the total amount of investment in solar-PV decentralised technologies per country (if all the mini-grid investments recommended using the analysis of the PV-DEI Index were undertaken). The overall investment costs are calculated by aggregating the costs of each PV mini-grid at national level [1]. In case of private investments approach, the PV-DEI index allowed to estimate the overall investment cost for each country, showing that for three-top PV-DEI countries the overall investment cost were of approximatively EUR 890 million for Ethiopia, EUR 550 million for Kenya and EUR 525 million investments for South Africa.

Table 2 summarises the market size for PV mini-grids which have been calculated for each country accounting for the proportion of population non-electrified versus total population per country and the potential market size for PV decentralised options (potential new costumers)

## 2 Experimental Design, Materials and Methods

**Fig. 4:**
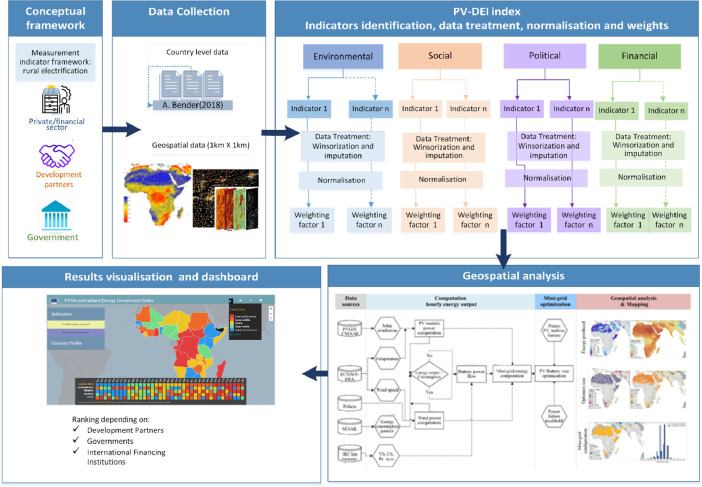
Analytical framework of the construction of the PV-DEI index approach.

The PV-DEI index composite indicator was built in accordance with the ‘best practice’ for composite indicator design outlined by the European Commission's guidance on composite indicators [24]. The structure of the Index was empirically tested, and improved in terms of accuracy and robustness whenever possible [1,24]. Fig. 4 illustrates the conceptual and analytical framework.

The quality of any composite indicator is determined by the quality of the base data used to populate the index, and the validity of the processes used in the construction of the index. Consequently, data selection was critical in determining the overall quality of the PV-DEI composite indicator. To ensure data sets used to construct the indicator were not selected based on convenience, and thus allowed to modify the structure of the PV-DEI index in a *post hoc* fashion based on observed data availability, the structure of the PV-DEI index was determined prior to data selection. This was done through an extensive review of the existing literature on the factors important for the direction of decentralised solar-PV investment. Data was then selected in accordance with the *a priori* specified structure PV-DEI index structure.

The search for relevant data proceeded through online search engine enquiries, in addition to more specific searches using resources provided by the World Bank [8,^10^,17], World Health Organisation [11], and the United Nations Development Programme [14]. The quality of the indicator data was assessed using a combination of criteria outlined by the OECD and the European Commission in the ‘Handbook on Constructing Composite Indicators’[24]. Thus, data-sets were relevant to the overall purpose of the PV-DEI Index, measured within an appropriate timeframe for the phenomenon of interest, appropriately sensitive to slight changes in this phenomenon, interpretable and complete with clear definitions of the items and/or populations studied, coherent across SSA countries, accurate and reliable (Table 1).

Overview:

The steps completed to ensure data was appropriate for use in the final composite indictor were as follows:

1. The indicator datasets were initially grouped according to the pre-defined conceptual framework.
2. The datasets were intensified to ensure they were comparable across countries. For example, by dividing by a country's population or other indicator-appropriate metric.
3. The indicators were checked for skew and kurtosis. In the COIN tool used for data processing data sets were considered skewed when skew was greater 2 and kurtosis was considered high if it was greater than 3.5 [24]
4. Data sets were winsorized when skew was greater than 2 and kurtosis was greater than 3.5
5. Countries missing more then 65% of data across the indicators were removed
6. Structural assessments (principal component assessments and correlational assessments) were conducted to investigate the underlying structure of the index.
7. Missing data was imputed using the MissForest package in R.
8. Structural assessments were re-run to ensure data-imputation had not significantly altered the underlying structure of the index.
9. Indicator data sets were normalised using the min-max method of normalisation.
10. In the DV-PEI index indicators were aggregated according to the weighting system devised in [1]. Using the raw data provided in this publication it is hoped stakeholders will be able to apply their own weights based on the importance they place on particular indicators.

INITIAL PROCESSING

Once the indicator data had been compiled, data sets were initially intensified following the recommendations of the COIN tool for composite indicator design provided by the European Commission [24]. Data intensification ensured data sets were comparable across countries with diverse population sizes, land areas, and natural resources. Data sets were also winsorized, again following the recommendations of the COIN tool for best practice in composite indicator design. This removed the negative impacts of potentially spurious outliers within data sets. Countries missing more then 65% of data across the indicators were removed from the analysis using the COIN tool.

STRUCTURAL ASSESSMENTS

Structural assessments were then undertaken to assess the underlying structure of the PV-DEI index. Correlational assessments were conducted using the COIN tool to ensure no two indicators within the same sub-pillar were highly correlated (high positive correlation: +0.5), rendering the use of one of them redundant. This was repeated to additionally ensure no indicators were negatively correlated with other indicators in their subpillar (high negative correlation: −0.5), which would have suggested an inconsistency between the indicators and what was being measured. The COIN tool operates through excel and no coding was required for the correlational assessments.

Principal component assessments were also conducted using the R function prcomp(), to ensure that indicator groupings were consistent with the structure of the underlying data. This resulted in the relocation of Indicator 48 which measured the removal of taxes and tariffs from the financial dimension, to the political pillar that focuses on the creation of a decentralised energy market. This remained in keeping with the conceptual framework of the political dimension. After the completion of the structural assessments the PV-DEI index went from 55 to 52 indicators, and indicator 48 was relocated to a different pillar.

Imputation of missing data

The imputation of missing data was conducted using two different popular methodologies for data imputation, each requiring a different package in R:

1. Implementation using a random forest algorithm (MissForest)
2. Multiple Imputation via Chained equations (MICE)

In both cases datasets had been intensified and winsorized, and countries missing greater than 65% of data across indicators had been removed. Categorical indicators had been removed and missing data for these was imputed separately using the mode of the region of Africa in which the country missing data was located. The MissForest() function in R was used first to generate the imputed datasets. The maximum number of iterations to be performed if the stopping criteria had not been met was set at 10. The number of trees to grow in each forest was set to 300. The final datasets were normalised using the min-max method and used to calculate PV-DEI index scores for comparison with the Mice () output in a sensitivity assessment documented in Fig. 6.

**Fig. 5:**
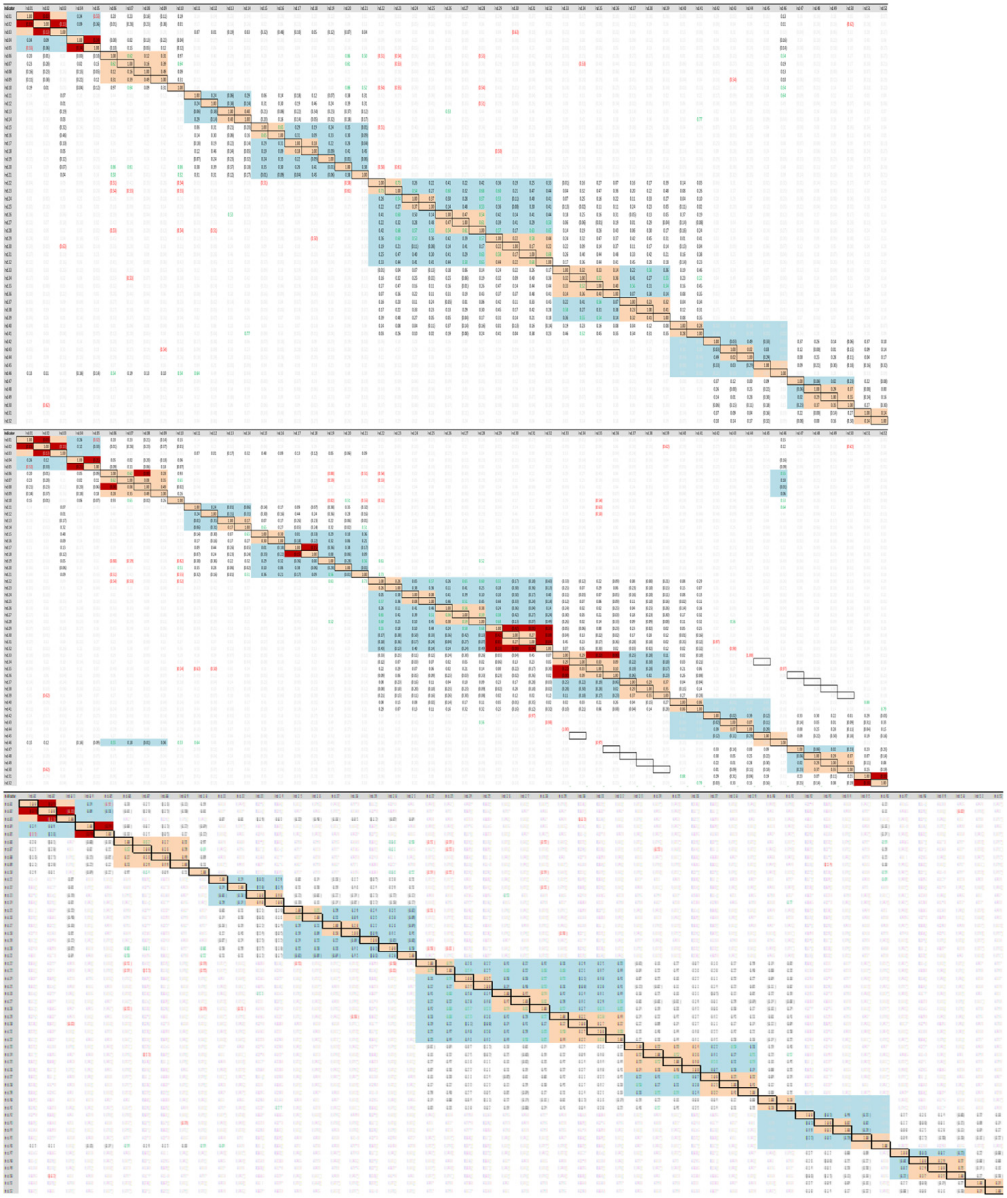
**A** Correlational assessments carried out in the COIN tool on the non-imputed data sets **B ¬** Correlational assessments carried out in the COIN tool showing results from one of the 5 MICE imputed data sets **C¬** Correlational assessments carried out in the COIN tool on the MissForest imputed data sets.

**Fig. 6:**
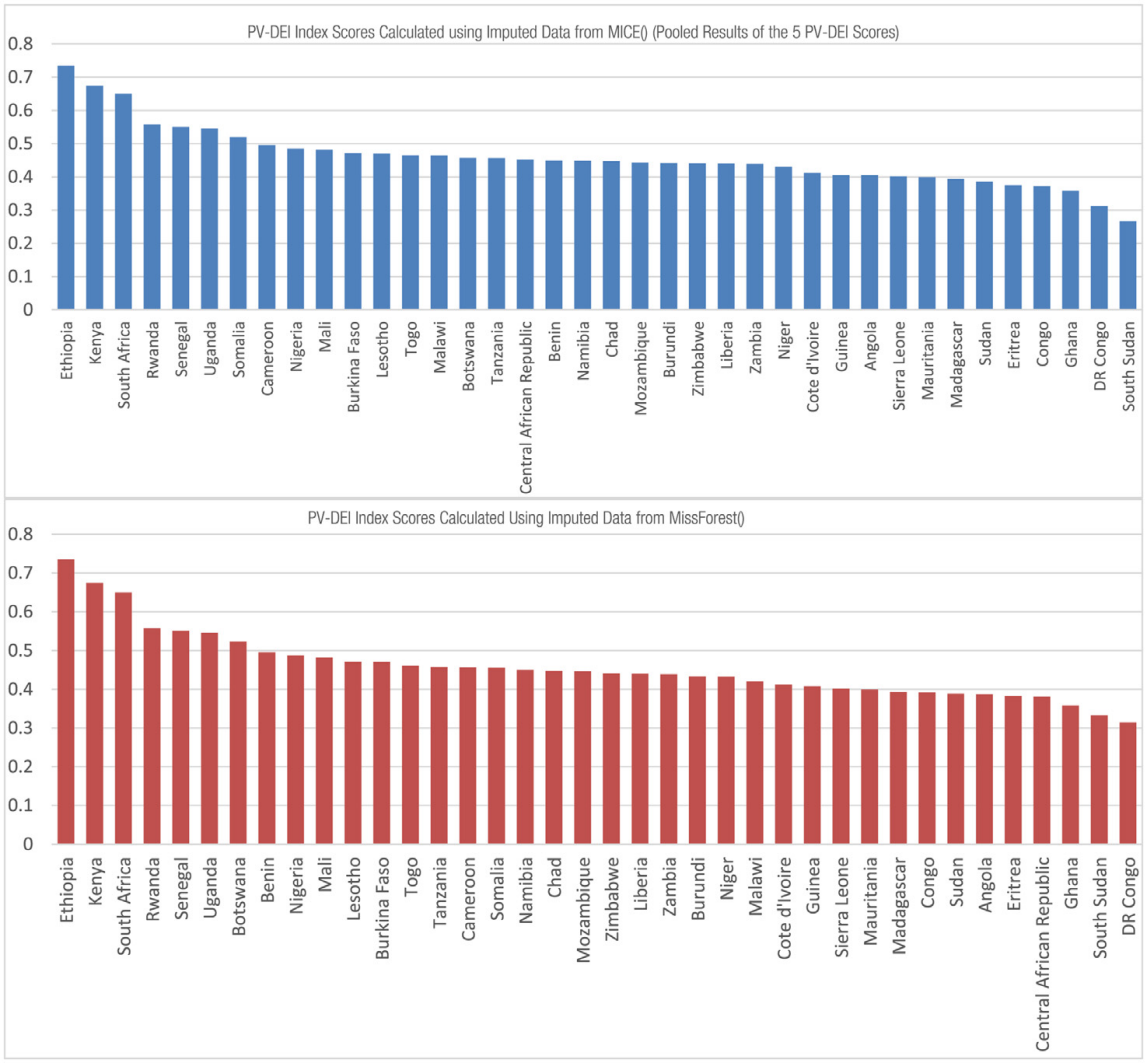
**A ¬**PV-DEI scores calculated using the pooled results of the 5 Mice() imputed datasets **.B** ¬PV-DEI Scores calculated using the MissForest() imputed data.

Following imputation using MissForest(), the MICE() function was used to generate 5 imputed data sets. Thus, the number of multiple imputations was set at 5, the method selected was a predictive mean matching model (PMM), the maximum number of iterations was set at 50. For each of the 5 data sets structural assessments were conducted (Fig. 5.C). The 5 datasets were normalised independently using the min-max method and used to calculate 5 separate composite indicator scores. The results of these were then finally pooled to create an average PV-DEI index score to enable a sensitivity assessment to be conducted comparing the MICE() and MissForest() methods of imputation (Fig. 6).

Comparison of MICE and MissForest – *Re*-running the Structural Assessments

When comparing the imputed data sets with the original data using the COIN tool, it was apparent that the MissForest method of imputation preserved the original relationships between the indicators to a greater extent than the MICE method of imputation (Fig. 5). Thus, the MissForest package in R was used to impute the missing data. The result of our sensitivity assessment comparing MICE and Miss forest imputation methods was in alignment with findings elsewhere that random forest techniques are more appropriate for imputing data in complex data sets as compared to multiple imputation using chained equations [1].

Comparison of MICE andMissForest– PV-DEI index scores

An additional sensitivity assessment was conducted to investigate how the ranking of countries within the PV-DEI index would alter if data was imputed using Mice() as compared to the MissForest() method. As demonstrated in Fig. 6 the ranking of countries within the PV-DEI was reasonably robust to the imputation method selected. With the exceptions of Cameroon, Somalia and the Democratic Republic of the Congo (DRC) – which all performed markedly better under Mice() imputation, most countries preserved their relative position between the two methods. Following the evidence provided by the structural assessments documented above, and relying on expert knowledge of the relative attractiveness of Cameroon, Somalia and the DRC for investment, PV-DEI scores obtained following the MissForest() method of imputation were used in the final index.

Normalisation

Following the imputation of missing data, the completed data sets were normalised using the min-max method of normalisation. This is the technique recommended as best practice within the COIN tool as it is able to preserve the shape of the data distribution for each indicator, and does not unduly rewards or punish exceptional indicator values. To investigate whether using an alternative popular normalisation technique, the Z-score transformation, would have significantly altered results on the PV-DEI index, sensitivity assessments were conducted comparing index results after normalisation using both min-max and Z-score normalisation techniques. The differences in scores were found to be slight as visualised in Fig. SI.6.

Aggregation

In the DV-PEI index indicators were aggregated according to the weighting system devised in ([1]). This was based on both expert knowledge obtained from an expert elicitation survey, and principal component assessments conducted at the level of the sub-pillars, see Fig. SI.7. However, the raw data provided in this publication is hoped to enable stakeholders to apply their own weights and thus create their own PV-DEI indices appropriate for their requirements.

Sensitivity Assessments

In addition to the sensitivity assessments documented here, additional sensitivity assessments were conducted to investigate the impact of data winsorization (Fig. SI.3) on PV-DEI scores.

## CRediT Author Statement

**Bender, A:** Conceptualization, Methodology, Validation, Investigation, Writing, Review & Editing; **Moner-Girona, M:** Conceptualization, Methodology, Investigation, Visualization, Writing, Review & Editing; **Becker, W:** Methodology, Investigation, Writing, Review & Editing; **Bódis, K:** Data processing, Geospatial modelling and analysis, Visualization; **Szabó, S:** Investigation, Writing, Review & Editing; **Kararach, A. G.:** Writing, Review & Editing. **Anadon, L. D,** Conceptualization, Writing, Review & Editing.

## Declaration of Competing Interest

The authors declare that they have no known competing financial interests or personal relationships which have or could be perceived to have influenced the work reported in this article.

*Disclaimer:* The views expressed are purely those of the authors and may not in any circumstances be regarded as stating an official position of the European Commission.

## Appendix Supplementary materials

Image, application 1
